# Angiotensin I-Converting Enzyme Inhibitor Derived from Cross-Linked Oyster Protein

**DOI:** 10.1155/2014/379234

**Published:** 2014-07-23

**Authors:** Cheng-Liang Xie, Jin-Soo Kim, Jong-Myung Ha, Se-Young Choung, Yeung-Joon Choi

**Affiliations:** ^1^Department of Seafood Science and Technology/Institute of Marine Industry, Gyeongsang National University, Gyeongnam-do 650-160, Republic of Korea; ^2^Department of Pharmaceutical Engineering, College of Medical and Life Science, Silla University, Busan 617-736, Republic of Korea; ^3^Department of Hygienic Chemistry, College of Pharmacy, Kyung Hee University, Seoul 130-701, Republic of Korea

## Abstract

Following cross-linking by microbial transglutaminase, modified oyster proteins were hydrolyzed to improve inhibitory activity against angiotensin-converting enzyme (ACE) inhibitory activity with the use of a single protease, or a combination of six proteases. The oyster hydrolysate with the lowest 50% ACE inhibitory concentration (IC_50_) of 0.40 mg/mL was obtained by two-step hydrolysis of the cross-linked oyster protein using Protamex and Neutrase. Five ACE inhibitory peptides were purified from the oyster hydrolysate using a multistep chromatographic procedure comprised of ion-exchange, size exclusion, and reversed-phase liquid chromatography. Their sequences were identified as TAY, VK, KY, FYN, and YA, using automated Edman degradation and mass spectrometry. These peptides were synthesized, and their IC_50_ values were measured to be 16.7, 29.0, 51.5, 68.2, and 93.9 *μ*M, respectively. Toxicity of the peptides on the HepG2 cell line was not detected. The oyster hydrolysate also significantly decreased the systolic blood pressure of spontaneously hypertensive rats (SHR). The antihypertensive effect of the oyster hydrolysate on SHR was rapid and long-lasting, compared to commercially obtained sardine hydrolysate. These results suggest that the oyster hydrolysate could be a source of effective nutraceuticals against hypertension.

## 1. Introduction

Hypertension affects about 70 million people in the USA, with an overlap in those suffering from cardiovascular disease [1], and it is also a major cause of death in Korea [2]. Blood pressure is regulated by the renin-angiotensin-aldosterone system (RAAS). Angiotensin I-converting enzyme (ACE) converts inactive decapeptide angiotensin I into octapeptide angiotensin II, raising the blood pressure in mammals [3, 4]. Synthetic ACE inhibitors such as Captopril, Lisinopril, Enalapril, and Fosinopril are pharmaceuticals used to treat hypertension. However, these drugs cause strong side effects, including coughing, skin rashes, and angioedema, whereas the ACE inhibitory peptides derived from food proteins are not associated with these side effects [5, 6]. ACE inhibitory peptides have been identified from various marine animals, such as squid [7], Alaskan pollock [8], tuna [9], Pacific hake [10], squid skin [11], skipjack roe [12], jelly fish [13], and ribbonfish backbone [14]. Several ACE inhibitory peptide products from food sources are currently on the market, such as Vasotensin, Valtyron, Calpis, and Evolus [15, 16].

Oysters are abundantly maricultured along the seashore of Tongyeong, Korea. The decline in export and consumption of fresh oysters promoted the search for a new application. ACE inhibitory peptides have been isolated and characterized from an oyster protein and from salt-fermented oyster sauce [17, 18]. New ACE inhibitory peptides might also be produced from oysters, depending on conditions including the kinds of protease used, hydrolysis temperature, and hydrolysis time. Oysters are a suitable protein source for the production of nutraceuticals against hypertension.

Transglutaminase (TGase; protein-glutamine *γ*-glutamyltransferase, EC 2.3.2.13) catalyzes an acyl-transfer reaction between the *γ*-carboxamide group of peptide-bound glutamine residues (acyl acceptors) and a variety of primary amines (acyl acceptors), including the *ε*-amino group of lysine residues in certain proteins [19]. TGase derived from a microbial source (microbial TGase, MTGase) is now commonly used for the treatment of food to improve flavor, appearance, and texture [20]. Protein functionality is altered by TGase-induced cross-linking [21, 22] which alters the heat-induced gelling ability of muscle proteins [23, 24]. It has been reported that the *ε*-(*γ*-glutamyl)lysine bond formed through protein cross-linking exhibits high resistance to proteolytic degradation [20]. Moreover, cyclic peptides with high affinity and specificity to biological targets have been produced by the cross-linking role of MTGase [25].

In the present work, we aimed to optimize the enzymatic hydrolysis of MTGase-cross-linked oyster protein, to purify and identify the small ACE inhibitory peptides from oyster hydrolysate prepared under optimized hydrolysis conditions, and to evaluate the antihypertensive activity of the oyster hydrolysate in vivo.

## 2. Materials and Methods

### 2.1. Materials

Oysters were purchased from a local market in October 2011 (Tongyeong, Korea). ACE enzyme (EC 3.4.15.1, Sigma A6778), pepsin (EC 3.4.23.1, 570 units/mg, Sigma P7125), trypsin (EC 3.4.21.4, 12,800 units/mg, Sigma T1426), N-Hisppuryl-His-Leu hydrate (HHL, Sigma H1635), and Captopril (Sigma C4042) were purchased from Sigma Chemical Co. (St. Louis, MO, USA). Several specific peptides (TAY, VK, KY, FYN, and YA) were synthesized at GL Biochem Ltd. (Shanghai, China). Sardine hydrolysate was purchased from Chosunmuyak Co. (Seoul, Korea). Alcalase 2.4 L (2.4 AU/g, endopeptidase,* Bacillus licheniformis*), Flavourzyme 500 MG (500 LAPG/g, endoprotease and exopeptidase,* Aspergillus oryzae*), Protamex 1.5 MG (1.5 AU/g,* Bacillus* protease, complex), and Neutrase 0.8 L (0.8 AU/g, endoprotease,* Bacillus amyloliquefaciens*) were obtained from Biosis Co. (Busan, Korea). MTGase (103 U/g) was obtained from Ajinomoto Co. (Tokyo, Japan). Acetonitrile and methanol were high-performance liquid chromatography (HPLC) grade. All other reagents were reagent grade. HiLoad Q-Sepharose, Superdex peptide, and Source 5RPC ST columns were purchased from GE Healthcare (Parsippany, NJ, USA). A Bondclone C18 column was purchased from Daiso Chemical Co. (Tokyo, Japan).

### 2.2. Preparation of Oyster Hydrolysates

**Figure 1:**
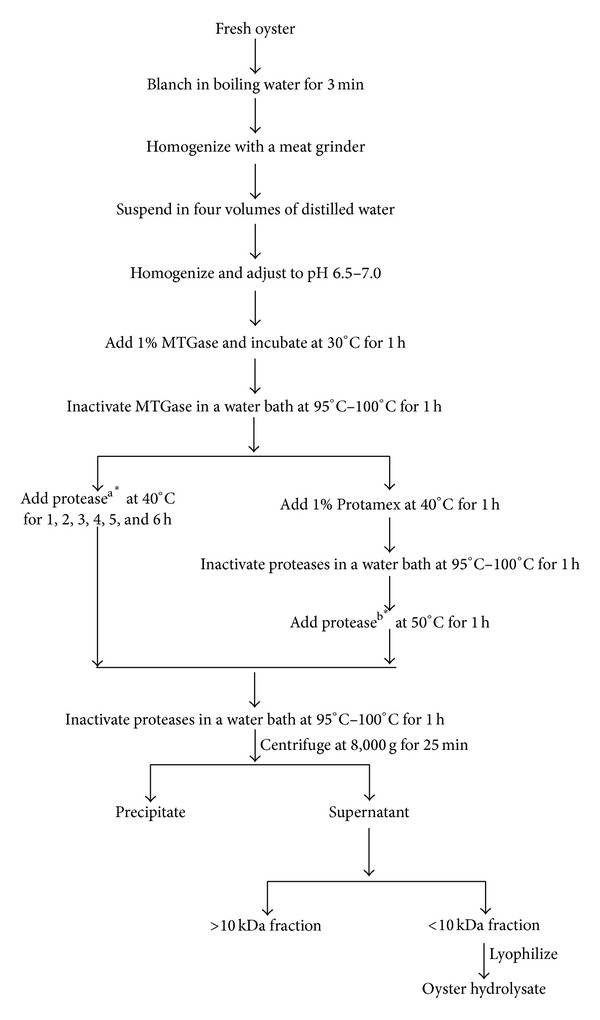
Scheme for preparation of the oyster hydrolysate. Protease^a^: the ratio of protease to fresh oyster was 1% for Alcalase, Flavourzyme, Neutrase, and Protamex, 0.1% for pepsin, and 0.05% for trypsin; protease^b^: the ratio of protease to fresh oyster was 1% for Alcalase, Flavourzyme, and Neutrase, 0.1% for pepsin, and 0.05% for trypsin; *: the pH for Alcalase, Flavourzyme, Neutrase, Protamex, and trypsin was 6.5–7.0; the pH for pepsin was 2.0.

Oyster protein was cross-linked by MTGase and the modified oyster protein was hydrolyzed by single proteases or a combination of the following six proteases: Alcalase, Flavourzyme, Neutrase, Protamex, pepsin, and trypsin. In detail, fresh oysters were treated in boiling water for 3 min to remove the fish odor and salt from the seawater and then homogenized using a meat grinder (M-12S, Hankook Fujee Industry, Hwaseong, Korea). The minced oyster was suspended in four volumes of distilled water and homogenized with a homogenizer (T-25 basic, Ika Works Inc., Wilmington, NC, USA). The suspension was adjusted to pH 6.5–7.0 with 1 M NaOH, after which 1% MTGase (by weight of fresh oyster) was added. The mixture was then incubated at 30°C for 1 h in a 5 L jar fermenter (Korea Fermenter Co., Seoul, Korea) with stirring at 150 rpm. After the reaction, MTGase was inactivated by immersion in a 95°C–100°C water bath for 1 h. To determine the optimum hydrolysis time and protease, the oyster protein was hydrolyzed with each protease for 1, 2, 3, 4, 5, or 6 h or by two-step hydrolysis using Protamex with the other five proteases, respectively, for 1 h in the same jar fermenter. The ratio of protease to fresh oyster was 1% for Alcalase, Flavourzyme, Neutrase, and Protamex, 0.1% for pepsin, and 0.05% for trypsin. After inactivating the proteases in a 95°C–100°C water bath for 1 h, the mixture was centrifuged (8,000 g, 25 min, Supra 22 K, Hanil Sci. Industry Co., Incheon, Korea), after which the supernatant was ultrafiltered with a 10 kDa membrane using a lab scale TFF system (Millipore Co., Billerica, MA, USA). The material with a molecular size < 10 kDa was lyophilized and stored at −20°C until use. The scheme and hydrolysis conditions for the preparation of oyster hydrolysate are shown in Figure 1.

### 2.3. ACE Inhibitory Activity Assay

ACE inhibitory activities of the oyster hydrolysates prepared with the different enzymatic hydrolysis conditions (Figure 1) were measured according to the method of Wu et al. [26], with slight modifications. A sample solution (20 *μ*L) was mixed with 225 *μ*L of ACE solution (0.025 units/mL), which was preincubated at 37°C for 10 min before adding 50 *μ*L HHL (2.5 mg/mL of 0.1 M borate buffer, pH 8.3 containing 0.3 M NaCl). The mixture was then incubated for 30 min at 37°C. The reaction was stopped by adding 75 *μ*L of 1 M HCl, and the mixture was centrifuged at 8,160 g (Micro 17TR, Hanil Sci. Industry Co., Incheon, Korea). The amount of hippuric acid (HA) in the supernatant was determined by reversed-phase HPLC (Shimadzu, Kyoto, Japan) on C18 (5 um, 4.6 × 250 mm). The IC_50_ value was calculated as the inhibitor concentration required for inhibition of 50% of the ACE activity. The percentage of inhibition of the enzyme activity was calculated as follows:

(1) $$\begin{array}{c} \%\;\text{inhibition}\;\text{activity}=\left[\frac{{\text{HA}}_{\text{control}}-{\text{HA}}_{\text{sample}}}{{\text{HA}}_{\text{control}}}\right]\times 100\%. \end{array}$$

### 2.4. Purification of the ACE Inhibitory Peptide

Oyster hydrolysate prepared at the optimized enzymatic condition was dissolved in 20 mM Tris-Cl, pH 8.0 buffer, and the ACE inhibitory fraction was eluted using a HiLoad Q-Sepharose column (16 × 100 mm). Elution was performed using a linear gradient system from solvent A (20 mM Tris-Cl, pH 8.0) to solvent B (20 mM Tris-Cl, pH 8.0 containing 0.75 M NaCl) over 100 min at a flow rate of 1 mL/min and detected at 254 nm. Every 2 mL fraction was collected, and ACE inhibitory activities were determined. Fractions with high ACE inhibitory activity were pooled and concentrated using an Amicon stirred cell with a 1 kDa membrane. The concentrated fractions (< 1 kDa) were loaded on a Superdex peptide column (10 × 300 mm) and eluted with 20 mM Tris-Cl, pH 7.5 at a flow rate of 0.5 mL/min. Detection was then carried out at 216 nm. Each 1 mL fraction was collected, and ACE inhibitory activities were detected. Fractions with high ACE inhibitory activity were dried completely in a speed vacuum concentrator (ScanSpeed 40, LaboGene Aps, Denmark) and applied to the reversed-phase column for further purification.

Active fractions were dissolved in 0.1% TFA in water and fractionated using the AKTA purifier system (GE Healthcare) with a Source 5RPC ST column (4.6 × 150 mm). Elution was performed using a linear gradient system from solvent A (0.1% TFA in water) to solvent B (0.1% TFA/60% ACN) over 90 min at a flow rate of 1 mL/min and detected at 216 nm. The column was equilibrated with solvent A, after which 50 *μ*L of sample was applied to the column and elution was carried as follows: two column volumes of solvent A, fourteen column volumes of solvent A-B gradient, four column volumes of solvent B, and four column volumes of solvent A. The purified ACE inhibitory peptides were dried in a speed vacuum concentrator for amino acid sequence identification.

### 2.5. Amino Acid Sequence Identification and Synthesis of Peptides

The sequence of peptides was analyzed by the Edman method using a 491 Protein Sequencer (Applied Biosystems, Foster City, CA, USA). The molecular mass of the peptides was determined by an AB SCIEX API 3200 QTRAP mass spectrometer (AB SCIEX, Framingham, MA, USA). The peptides were subsequently synthesized using the solid-phase method at GL Biochem Co. (Shanghai, China). The purities of the synthesized peptides were >95.8%, confirmed by reversed-phase HPLC analysis.

### 2.6. ACE Inhibitory Activity and HepG2 Cell Toxicity of the Synthetic Peptides

The ACE inhibitory activities of the synthetic peptides were measured, and the cell toxicity was evaluated by cell viability testing on the hepatocyte cell line HepG2. HepG2 cells were cultured in MEM medium containing 10% fetal bovine serum. Ninety-six well plates containing 1 × 10^4^ cells per well were incubated at 35°C for 24 h under 95% humidity and 5% CO_2_. After incubation, the synthetic peptides were added (final concentration, 200 *μ*g/mL) and cells were incubated under the same conditions for an additional 24 h. Cell growth was assessed with the CellTiter 96 Aqueous One Solution Cell Proliferation Assay kit (Promega, Madison, WI, USA) and absorbance was determined with a microplate reader (Perkin Elmer 1420, VICTOR X Multilabel Plate readers, Waltham, MA, USA) at 490 nm. Cell viability was calculated as the percentage of absorbance of the synthetic peptide-treated groups compared with that of the untreated group (100% viability).

### 2.7. Animal Studies

Spontaneously hypertensive rats (SHR) with systolic blood pressure (SBP) between 170 and 190 mmHg and original strain Wistar Kyoto rats (WKR) with SBP between 100 and 125 mmHg were purchased from Harlan, USA (Indianapolis, USA). All rats were treated in accordance with Kyung Hee University guidelines for the care and use of laboratory animals. The animals were individually housed in stainless steel cages and adapted to 23 ± 1°C and humidity of 55 ± 5% under a 12 h light-dark cycle. The 12-week-old rats were fed a pelletized commercial chow diet for a period of 1 week after arrival and then randomly divided into five groups: untreated SHR control group (*n* = 3); normal WKR control group (*n* = 3); oyster hydrolysate-treated SHR group (100 mg/kg body weight, *n* = 3); sardine hydrolysate-treated SHR group (positive control, 100 mg/kg body weight, *n* = 3); and Captopril-treated SHR group (positive control, ACE inhibitor; 8 mg/kg body weight, *n* = 3). After intragastric administration of the samples using Sonde, SBP of the rats was measured by a Coda noninvasive blood pressure system (Kent Scientific Corporation, Baltimore, USA) using the tail-cuff method after prewarming for 30 min in an environmental chamber of 32°C at 0, 3, 6, 9, 12, and 24 h. Subsequent measurement of blood pressure was expressed as a percentage of that at the initial time.

### 2.8. Statistical Analysis

Data were expressed as the mean with standard deviation of triplicate determinations. Analysis of variance was carried out by the Tukey HSD test using the JMP 10 package (SAS Institute, Carry, NC). Probability values less than 5% (*P* < 0.05) were considered statistically significant.

## 3. Results and Discussion

### 3.1. Comparison of Protease Treatments for Preparation of the Oyster Hydrolysate

**Figure 2:**
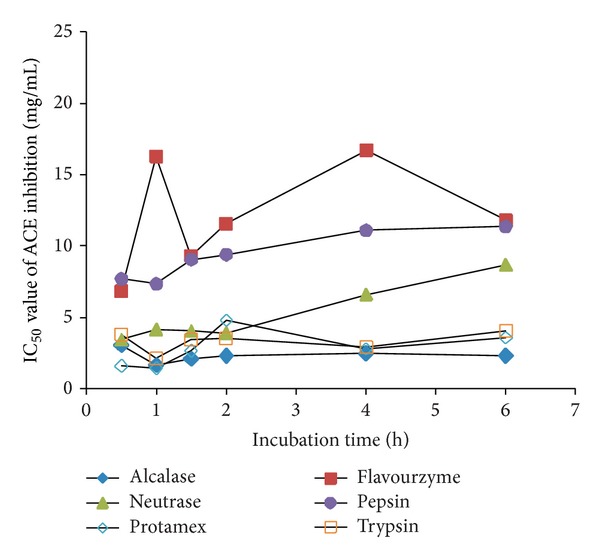
The IC_50_ values of ACE inhibition of the oyster hydrolysates according to proteases used and hydrolysis time.

The IC_50_ values of ACE inhibition of the oyster hydrolysate differed greatly with the variation of proteases and hydrolysis time. The Protamex-treated hydrolysate showed the highest ACE inhibitory activity when hydrolyzed at 40°C for 1 h, as demonstrated by the lowest IC_50_ values of 1.49 mg/mL (Figure 2). In previous studies, the most potent ACE inhibitory activities were found for Alcalase-treated squid gelatin [7], thermolysin-treated bonito muscle [27], pepsin-treated tuna dark muscle [9], and Protamex-treated hard clam meat [28]. These collective results suggest that the ACE inhibitory activity of a hydrolysate depends on the type of protease and the protein used as the substrate.

**Figure 3:**
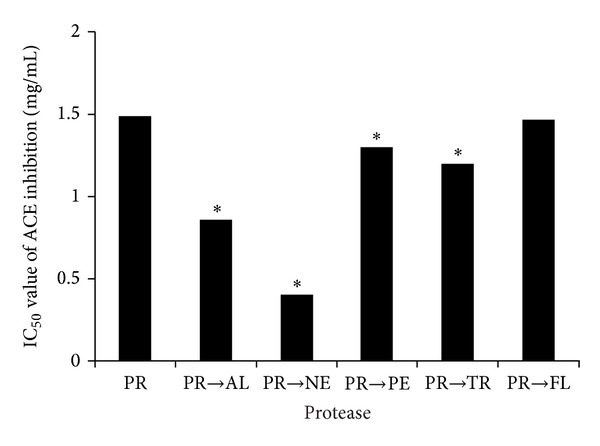
The IC_50_ values of the ACE inhibition of the oyster hydrolysate after two-step hydrolysis, with two proteases. PR, Protamex; AL, Alcalase; NE, Neutrase; PE, pepsin; TR, trypsin; and FL, Flavourzyme. The asterisks above bars denote statistical significance compared to PR (*P* < 0.05).

The ACE inhibitory activity was most improved by two-step hydrolysis with Protamex and Neutrase, demonstrating higher activities than hydrolysates prepared with one-step hydrolysis and two-step hydrolysis with the other proteases (Figure 3). In a previous study by Jang and Lee [29], the highest ACE inhibitory activity of an enzymatic hydrolysate from sarcoplasmic protein of beef resulted from the combination of thermolysin and proteinase A. Moreover, gelatin hydrolysate from the sequential treatment of sea cucumber with bromelain and Alcalase also showed high ACE inhibitory activity, with an IC_50_ value of 0.35 mg/mL [30]. Based on the results above, the two-step hydrolysis with Protamex and Neutrase was chosen as the optimum condition to prepare the oyster hydrolysate.

### 3.2. Purification of ACE Inhibitory Peptides

**Figure 4:**
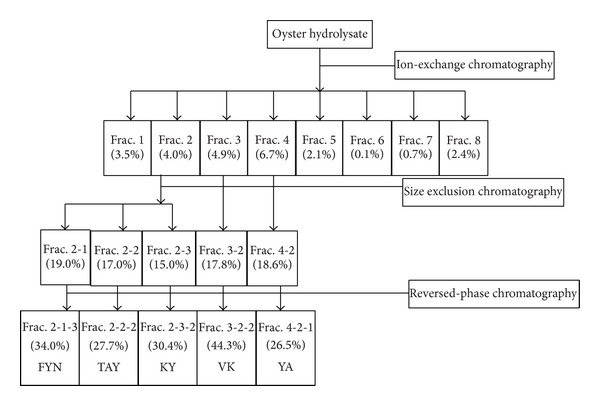
Purification scheme of the oyster hydrolysate and ACE inhibition activity of the fractions from ion-exchange, size exclusion, and reversed-phase chromatography. The numbers in parenthesis presented the ACE inhibitory activity of the fractions.

**Figure 5:**
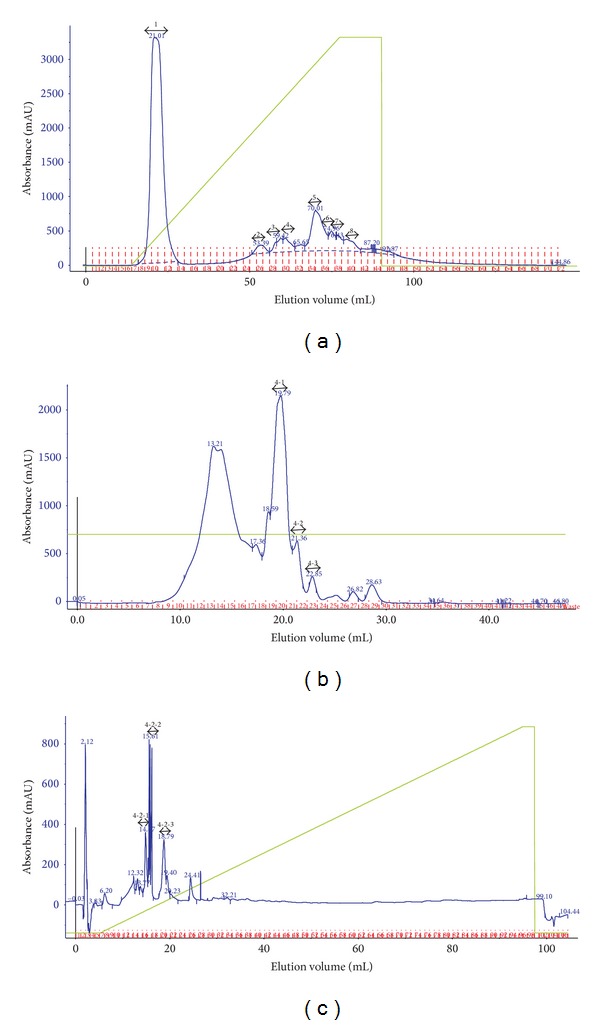
Separation procedures of ACE inhibitory peptides by ion-exchange (a), size exclusion (b), and reversed-phase chromatography (c). Ion-exchange chromatography was performed with a linear gradient of 20 mM Tris-Cl containing 0.75 M NaCl, pH 8.0 on a HiLoad 16/10 Q-Sepharose column (16 × 100 mm) for 100 min at a flow rate of 1 mL/min. Size exclusion chromatography was performed with 20 mM Tris-Cl, pH 7.5 on a Superdex peptide (10 × 300 mm) column at a flow rate of 0.5 mL/min. Reversed-phase chromatography was performed on a Source 5RPC ST column (4.6 × 150 mm). Eluent A consisted of 0.1% TFA/water (v/v), and eluent B was 0.1% TFA/60% acetonitrile (v/v) with a linear gradient for 90 min at a flow rate of 1 mL/min.

The oyster hydrolysate prepared at the optimum condition was separated into eight fractions using a Q-Sepharose ion-exchange column. Relatively high ACE inhibitory activities were observed in three fractions: 2, 3, and 4 (Figure 4). A typical chromatogram of the purification procedure is shown in Figure 5. ACE inhibitory activities of these three fractions were 4.0%, 4.9%, and 6.7%, respectively. The three active fractions were then divided further (Frac. 2-1, 2-2, and 2-3; Frac. 3-1 and 3-2; and Frac. 4-1, 4-2, and 4-3, resp.). Among these, five fractions (Frac. 2-1, 2-2, 2-3, 3-2, and 4-2) had high ACE inhibitory activities, in the range of 15.0%–19.0%. The pooled ACE active fractions from size exclusion chromatography were further purified by a Source 5RPC ST reversed-phase column. Five fractions with high ACE inhibitory activity were obtained: Frac. 2-1-3, 2-2-2, 2-3-2, 3-2-2, and 4-2-1, having increased activities in the range of 26.5%–44.3%.

### 3.3. Amino Acid Sequence Identification and Toxicity of ACE Inhibitory Peptides

**Figure 6:**
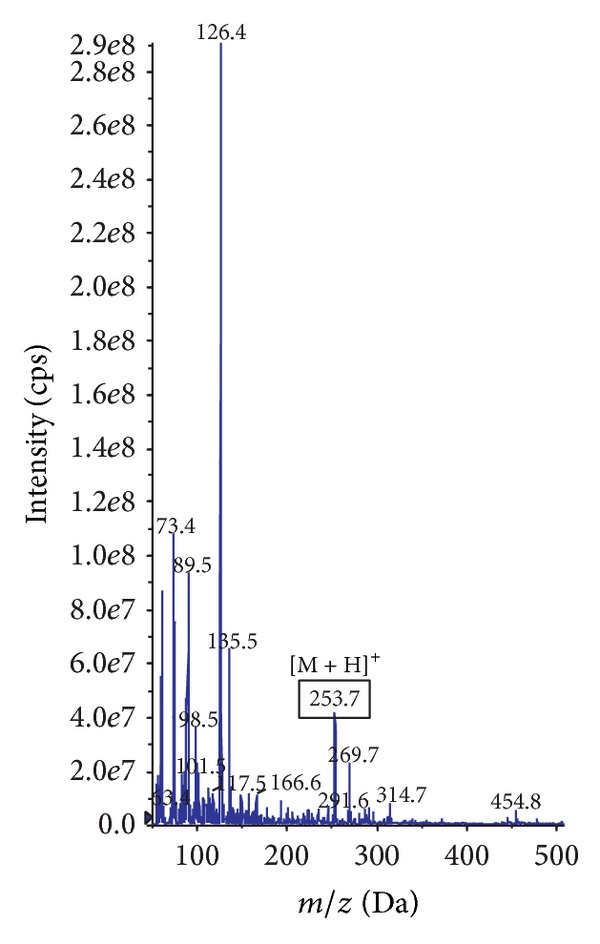
Mass spectrum (MS) of the pool named Frac. 4-2-1 (YA) from reversed-phase chromatography. The *m*/*z* value of [M + H]^+^ for YA was 253.7. cps: counts per second.

To identify the purified peptides, the active fractions (Frac. 2-1-3, 2-2-2, 2-3-2, 3-2-2, and 4-2-1) were subjected to automated Edman degradation and mass spectrometry. Manual analysis yielded the peptides sequences as FYN (442.5 Da), TAY (353.4 Da), KY (309.4 Da), VK (245.3 Da), and YA (252.3 Da), respectively. The mass spectra of Frac. 4-2-1 (YA) are shown in Figure 6 as an example; the *m*/*z* value of [M + H]^+^ (253.7 Da) matched well with the molecular weight of YA, 252.3 Da.

As a final step, the identified peptides of interest were synthesized using the solid-phase method. The IC_50_ values of the synthetic peptides TAY, VK, KY, FYN, and YA were subsequently measured to be 16.7, 29.0, 51.5, 68.2, and 93.9 *μ*M, respectively (Table 1). Cell viabilities in the HepG2 cell line ranged from 103.8 ± 7.7% to 117.5 ± 15.1% after treatment with the synthetic peptides copied from those in the oyster hydrolysate up to a concentration of 200 *μ*g/mL, compared to treatment with deionized water as a control (Table 1). These results suggested that the synthetic ACE inhibitory peptides had no toxicity for HepG2 cell line.

The molecular weights of the peptides were in the range of 200 Da to 500 Da, and all were composed of two or three amino acid residues. This was in compliance with our goal for the purification of small ACE inhibitory peptides from oyster hydrolysates. Because biological barriers demonstrate high diffusive resistance towards the uptake of large molecules, suitable carrier systems rarely exist. The absorption or permeation of molecules, having more than 5 H-bond donors, 10 H-bond acceptors, or a molecular weight greater than 500 Da, has been shown to be poor [31]. Pihlanto-Leppälä et al. [32] found that ACE inhibitory activity was higher in fractions having a molecular weight less than 1 kDa, which coincided with our results.

Moreover, the five peptides identified in this study all contained hydrophobic amino acid residues. Cheung and Li-Chan [33] reported that a fraction of shrimp hydrolysate which contained many hydrophobic residues showed strong ACE inhibitory activity. The results of Pripp et al. also showed a positive relationship between having a hydrophobic amino acid in the C-terminal position and the ACE inhibition of peptides [34].

### 3.4. Antihypertensive Activity of the Oyster Hydrolysate In Vivo

**Figure 7:**
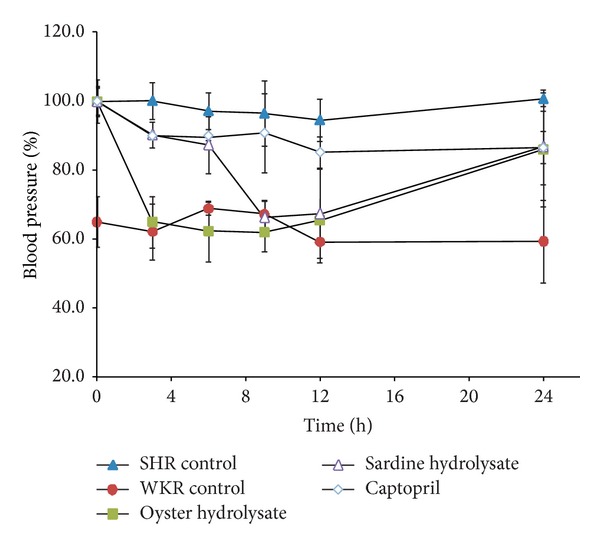
The SBP change of the test groups over time, compared with the initial SBP of each group, respectively; oyster hydrolysate, 100 mg/kg; sardine hydrolysate, 100 mg/kg; and Captopril, 8 mg/kg.

Antihypertensive activities of oyster hydrolysate were evaluated by measuring the SBP of SHR at 0, 3, 6, 9, 12, and 24 h after oral administration (Figure 7). SHR were developed by Okamono and Aoki by selective breeding of WKR with high blood pressure, while the normotensive WKR were employed as control for SHR [35]. The SHR model has been extensively used to investigate antihypertensive drugs in vivo [17, 36]. There was no significant change of SBP during 24 h for either the SHR or WKR controls. The SBP of the SHR significantly decreased 3 h after administration of the whole oyster hydrolysate and the antihypertensive activity lasted up to 12 h after administration, compared to the WKR control (*P* < 0.05), which had initial SBP in the range of 62.0 ± 5.6% to 65.5 ± 6.2%. Sardine hydrolysate and Captopril were used as positive controls. The SBP of the SHR was significantly lowered 9 h after administration of the sardine hydrolysate and the antihypertensive activity continued up to 12 h after administration compared to the WKR control (*P* < 0.05). However, the maximum decrease in SBP by Captopril was observed 12 h after administration, which was 85.3 ± 4.5% of the initial SBP (Figure 7). Captopril had a slight effect on the SBP of SHR but the antihypertensive activity was not significant (*P* = 0.33), which may be due to the small dosage administered [36]. Compared to sardine hydrolysate, the antihypertensive effect of the oyster hydrolysate on SHR was rapid and long-lasting. These results indicate that the whole oyster hydrolysate can be used as a source of nutraceuticals for antihypertension.

## 4. Conclusions

This study demonstrated that the ACE inhibitory activity of the MTGase-cross-linked oyster protein can be significantly improved by a two-step hydrolysis with Protamex and Neutrase. Five ACE inhibitory peptides were purified and identified from the oyster hydrolysate using ion-exchange, size exclusion, and reversed-phase chromatography. The oyster hydrolysate showed high antihypertension effect in vivo compared to sardine hydrolysate. It is, thus, possible to produce natural and effective antihypertensive products from oyster through enzymatic hydrolysis. Such oyster hydrolysate can be utilized as a nutraceutical or functional food, and the purified ACE inhibitory peptides have the potential for use as lead compounds of an antihypertension drug. Further work, including analysis of the physicochemical properties and quality control parameters for the hydrolysate, is in progress and will be reported.

## Figures and Tables

**Table 1:** IC_50_ values of ACE inhibition of the synthetic peptides and cell viability of HepG2 cell line treated with the synthetic peptides.

Peptide	IC_50 _(*μ*M)	Cell viability (%)^a^
TAY	16.7	105.1 ± 1.9
VK	29.0	117.5 ± 15.1
KY	51.5	118.2 ± 7.0
FYN	68.2	109.5 ± 3.0
YA	93.9	103.8 ± 7.7

^a^Deionized water was used as control; the final concentration of the synthetic peptides was 200 *μ*g/mL.
